# A cross-sectional workforce survey of three traditional and complementary medicine professions in Ontario, Canada

**DOI:** 10.1371/journal.pone.0250223

**Published:** 2021-05-13

**Authors:** Nadine Ijaz, Sandy Welsh, Qi Zhang, David Brule, Heather Boon

**Affiliations:** 1 Leslie Dan Faculty of Pharmacy, University of Toronto, Toronto, ON, Canada; 2 Department of Sociology, Faculty of Arts and Science, University of Toronto, Toronto, ON, Canada; 3 Institute of Health Policy, Management and Evaluation, University of Toronto, Toronto, ON, Canada; Endeavour College of Natural Health, AUSTRALIA

## Abstract

**Background:**

Workforce studies about traditional and complementary medicine (T&CM) occupations in industrialized countries are scant; and, these occupations’ position within the broader occupational workforce remains unclear. This study aims to address these gaps using a comparative approach.

**Methods:**

Naturopaths, traditional Chinese medicine (TCM) / acupuncture practitioners, and homeopaths in Ontario, Canada were surveyed regarding their demographics, practice characteristics and self-reported income. Results were compared with parallel data from within and outside of Ontario.

**Results:**

**S**tudy response rate: 23.3% (n = 1205). While predominantly female (57.9%), Ontario’s TCM/acupuncture profession was less feminized than the naturopathic (77.1%) and homeopathic (78.3%) groups. Naturopaths were significantly younger than, and reported fewer years of clinical experience than, the other two groups. About half of TCM/acupuncture practitioners, and almost one-third of homeopaths had trained outside of Canada, predominantly in East and South Asia, respectively. More TCM/acupuncture practitioners (58.9%) and homeopaths (57.6%) had multilingual clinical practices than naturopaths (19.1%). Homeopaths worked fewer hours and saw fewer patients per week than the other occupations. Self-reported mean incomes varied across groups, with naturopaths earning more on average ($63,834, SD $57,101) than did TCM/acupuncture practitioners ($45,624, SD $44,081) or homeopaths ($29,230, SD $41,645). Holding other variables constant, internationally-trained practitioners reported earning one-third less than their Canadian-trained counterparts.

**Discussion & conclusions:**

Study findings echo occupationally-specific data from other industrialized jurisdictions; and, affirm that different T&CM occupations have distinctive demographic and practice characteristics. The demographic makeup of Ontario’s TCM/acupuncture and homeopathy occupations suggests a role for these groups in delivering culturally-responsive care within Asian ethnic communities. T&CM practitioner incomes, in particular for internationally-trained practitioners, fell below the provincial population income median, and in many cases below the poverty line. T&CM occupations’ relative socio-political marginality may be impacting clinicians’ ability to earn a viable living.

## Introduction

Demand for the services of traditional and complementary medicine (T&CM) practitioners continues to increase across industrialized countries [1–5], though biomedicine remains the politically dominant form of health care worldwide [6]. Traditional and complementary medicine occupations, which include such diverse groups as chiropractors, naturopaths and homeopaths, herbalists, and practitioners of traditional medicine systems like Chinese medicine and Ayurveda [7]. Several nations have advanced the World Health Organization (WHO)’s safety-focused recommendation that governments regulate T&CM practitioner groups, whose health care approaches remain poorly integrated into conventional care in many countries [7]. Concurrently, T&CM practitioner groups in many places are taking steps to professionalize, as they aim to secure a position within their nations’ established healthcare workforces [8–10].

Workforce data represent an important resource for planners and policy makers as they make decisions about how to govern particular healthcare occupations within a broader health systems ecology [11]. However, such data about T&CM occupational groups remain scant across many jurisdictions, owing partly to the incomplete inclusion or omission of marginal occupations and emerging professions within government surveys [12, 13]. This gap is especially salient given that 88% of surveyed WHO member states have identified “lack of research data” as a policy-making barrier in the T&CM occupational field [7].

Prior workforce studies suggest that distinct T&CM practitioner groups may differ considerably from one another [14], united perhaps more by their sociocultural marginality than their demographic and practice patterns [7].Three key gaps remain in the literature with respect to the features and positionalities of such occupational groups within the broader ecology of health professions:

1. It remains unclear to what degree workforce characteristics for particular T&CM occupations may be extrapolated from one jurisdiction to another, given the range of local factors that may impact such traits.
2. Existing research provides little insight as to how T&CM practitioner characteristics—on the whole, and for particular occupations—may compare with conventional biomedical occupational characteristics within a given jurisdictional setting. In other words, while recognizing that T&CM practitioner groups are occupationally distinct, are there workforce traits shared across T&CM occupations that are less prominent within non-T&CM healthcare occupations?
3. Finally, while previous studies have documented the professionalization challenges faced by T&CM occupational groups, we are unaware of prior research examining what types of workforce factors maybe associated with greater professional success (i.e. higher income) for T&CM clinicians.

The present study aims to address these gaps by reporting on a cross-sectional online survey of three T&CM occupational groups in the province of Ontario, Canada. The survey was undertaken in 2018–19, shortly after all three groups–naturopaths, traditional Chinese medicine (TCM) / acupuncture practitioners, and homeopaths–were granted statutory self-regulatory status under the province’s Regulated Health Professions Act, which also governs the province’s physicians and other allied (non-physician) health care professionals [8]. To date, no detailed workforce data have been published about the studied occupations.

As shown in Table 1, the three occupations under study represent T&CM practitioner groups about which prior workforce studies have been undertaken across other industrialized jurisdictions (i.e., North America, Europe and Australia/New Zealand). Overall, these studies provide a comparative baseline for the three studied groups with respect to: demographic features (gender, age, years in practice, country of training, languages spoken); practice patterns (hours worked and patients per week); and, annual income. Furthermore, until 2013, the Ontario government published similar annual workforce data (with the exception of income) for its allied health professions, which at the time included the T&CM professions of chiropractic and registered massage therapy [29]. The research team took these inter- and intra-jurisdictional datasets as valuable comparators to help meet the study aims. We turn now to the study’s methods.

**Table 1:** Workforce profiles of naturopaths, TCM/acupuncture practitioners, and homeopaths across industrialized countries.

	Naturopaths	TCM/Acupuncture Practitioners	Homeopaths
	[Year, country]	[Year, country]	[Year, country]
**Female**	86.7% [2020, Australia] [15] 82.5% [2019, Switzerland] [14] 80.5% [2017, Germany] [16] 91%^a^ [2015, Australia] [17] 59% [2006, Canada] [18] 70% [2005, Australia] [19] 57–58%^b^ [2004, USA] [20]	57% [2016, Australia] [21] 61% [2019, Switzerland] [14] 71.3% [2017, USA] [22] 70% [2013, USA] [23] 69.7% [2013, USA] [24]	93% [2017, New Zealand] [25]
**Age,** mean (SD)	45.5 (10.4) [2020, Australia] [15] 52.6 (10.8) [2019, Switzerland] [14] 52 (9.5) [2017, Germany] [16] 42 (10.9) [2006, Canada] [18] 44 (10.4) [2005, Australia] [19] 43.6–44.1 (8.7–9.2)^b^ [2004, USA] [20]	50.6 (8.9) [2019, Switzerland) [14] 49 [2013, USA] [23] 54.8% of clinicians aged 40–59 [2010, USA] [26]	54% of clinicians aged 45–54 [2017, New Zealand] [25]
**Years in practice,** mean (SD)	6.6–8.2 [2020, Australia] [15] 12.7 (7.5) [2019, Switzerland] [14] 8.7 (8.5) [2006, Canada] [18] Median 9, 7^b^ [2004, USA] [20]	13.1 (7.9) [2019, Switzerland) [14] 11 [2013, USA] [23]	12.6 (7.8) [2017, New Zealand] [25]
**Trained outside country of practice**	18.6% [2019, Switzerland] [14]	29.5%; 6.8% China [2019, Switzerland) [14] 20.6% Chinese-born [2016, Australia] [21] 20% East Asian; twice that (narrative, account, no %) in California [USA] [26]	No data available.
**Multilingual clinic (***languages***)**	No data available.	~20%Chinese[2016, Australia] [21] 36.9% Chinese; 21.4% Korean; 8.5% Spanish [2015, California, USA] [27]	No data available.
**Hours / week**, mean (SD)	41 [2020, Canada] [28] 44 [2020, USA] [28] 17.6 (18.2) [2020, Australia] [15] 27.8 (14.4) [2019, Switzerland] [14] 25.4 (15.2) [2017, Germany] [16]	24.6 (13.4) [2019, Switzerland) [14] 40.9% >30 hrs/week [2010, USA] [26]	14 (9.2) clinical consultations + 5 (6.7) clinic management [2017, New Zealand] [25]
**Patients / week** mean (SD)	12.2 (10.2) [2020, Australia] [15] 13.6 (14.3)^c^ [2019, Switzerland] [14] 20.4 (20.3) [2017, Germany] [16] 32.7 (18.9), 30.5 (24.7)^b^ [2004, USA] [20]	20.8 (18.2)^c^ (2019, Switzerland) [14] 20 [2016, Australia] [21]	6.3 (7.3)^c^ [2017, New Zealand] [25]
**Income**, mean (SD), *median*	$83,973, *$60*,*000* [2020, Canada] [28] $95,642 USD, *$70*,*000* [2020, USA] [28]	*Between $40*,*000 and $50*,*000 USD* [2017, USA] [13]	No data available

^a^Study included both naturopaths and Western herbalists

^b^Study included two sets of surveyed clinicians, both sets are represented here

^c^Data reported in the original study as patients per *month*, divided by 4 here for comparison.

## Methods

The University of Toronto’s Research Ethics Board granted approval for the conduct of this study, funded by Canadian Institutes of Health Research. In late 2018 and early 2019, the authors used a census-style, online methodology to survey all Ontario TCM/acupuncture practitioners, naturopathic doctors, and homeopaths shortly after each of these groups had achieved statutory self-regulatory status in the province.

### Design

Following the Tailored Design method, the authors constructed three survey instruments–one for each of the occupational groups under study. The instruments shared fifty-two common questions meant to ask respondents about their:

- Demographic backgrounds (including gender, age, years in practice, country of training, and languages spoken in clinical practice);
- Practice characteristics (including hours worked per week, patients seen per week, professional practice site, provincial region of practice); and
- Annual self-reported, pre-tax income.

Additional survey items (not analysed in the present work) interrogated: T&CM practitioner views about their occupations’ recent statutory self-regulation under Ontario’s Regulated Health Professions Act; a series of occupationally-specific questions were included to gather data about each profession’s unique characteristics; and, a final open-ended question. Naturopathic and homeopathic surveys were constructed in English only, whereas Chinese-language translations (to both standard and simplified Chinese) of the English language TCM/acupuncture practitioner survey were also prepared. The survey instrument was designed also to measure the association of key variables (income, attitudes toward regulation) with characteristics of each practitioner group.

The research team consulted with nine registered practitioners across each of the professional groups (three TCM/acupuncture practitioners, three naturopaths and three homeopaths) to pre-test and gather feedback about the common and occupationally-specific questions. All pre-testers were independent from the research team. The team reviewed pre-testing feedback and incorporated relevant changes to the survey instruments. While this pre-testing procedure served as an informal validation process for the survey items, the survey instrument did not undergo formal validation.

### Sample compilation

The study team took a multi-pronged approach to gathering names and contact information for prospective survey participants, as described below.

Beginning in late 2017, the team used the provincial online registers for each of the professions under study to gather the names of all actively- and previously-registered members in good standing of the three studied occupational groups (N = 5143: TCM, n = 2953; Naturopaths, n = 1663; and Homeopaths, n = 527). These online registers are available to the public without any stipulations that would have impeded our use of their contents for research purposes.

The research team’s goal was to obtain contact information, preferably email addresses, for all named practitioners. In 2017–18, the public provincial registers for each profession included registrants’ street mail addresses but no email addresses. However, the team had accessed email addresses for a substantial number of Ontario’s TCM/acupuncture registrants in 2014 (n = 2921), during a period when the profession’s regulator had (in alignment with other provincial regulators) included email addresses on their public register. To fill the gaps in the team’s email contact lists across all three professional groups, extensive public internet searches were conducted. Where no email addresses were found, public internet searches were undertaken to identify practitioners’ street addresses.

### Recruitment and survey launch

The surveys launched in late 2018 and early 2019, using the Qualtrics online platform to facilitate anonymous, voluntary participation over an eight-week response period. All prospective participants for whom the team had email addresses received email invitations at launch, with reminders sent one, three and five weeks following initial recruitment; lettermail invitations were sent to those for whom only street addresses were available, followed by postcard reminders mailed two weeks following launch.

In total, after accounting for incorrect contact information (i.e., email bounce-backs and returned lettermail), the study team was able to successfully send recruitment letters to a total of 5170 prospective participants. The recruitment sample represented 2991 TCM/acupuncture practitioners (email: 2844; lettermail: 147), 1518 naturopaths (email: 1354; lettermail: 164), and 538 homeopaths (email: 446; lettermail: 28). As per the approved Ethics Board protocol, participants were advised in writing that their voluntary participation in the survey would be constituted as their informed consent to participate in the study.

### Analysis

To permit appropriate comparisons with other regulated professionals in the Ontario, Canada context, the present analysis includes only those survey respondents who self-identified as being actively registered with their respective provincial regulatory body.

Demographic, training and practice characteristics were summarized by employing standard descriptive analyses (e.g., percentage for categorical variables, means/standard deviations for continuous variables). Pearson’s chi-square test was used to compare categorical characteristics of practitioners across the three professions. Means and medians of continuous variables across the three professions were compared using analysis of variance and the nonparametric median test. In addition, two-by-two comparisons were conducted among the three professions for all characteristics.

The authors examined respondents’ self-reported incomes using linear regression. The characteristics of the dependent variable—income—and all independent variables can be found in Table 2. The logarithmic form of income, hours worked per week, and patients seen per week were used to capture the nonlinear property of the association. All analyses were performed using Stata/SE 15.1.

**Table 2:** Demographics, practice characteristics and annual self-reported income.

	Naturopaths	n	TCM/ Acupuncture Practitioners	n	Homeopaths	n	p-values
**Demographics**							
Gender (% female)	77.1%	279	57.9%	439	78.3%	115	<0.001
Age, Mean (SD)	42.5 (10.2)	264	51.5 (9.8)	417	53.1 (10.2)	112	<0.001
Years in clinical practice, Mean (SD)	11.3 (8.5)	310	17.9 (10.1)	489	14.4 (9.8)	128	<0.001
Trained outside of Canada	2.9%	313	50.4%	502	30.9%	136	<0.001
Multilingual practice	19.9%	282	58.9%	470	57.6%	125	<0.001
**Practice Characteristics**							
Full-time (30+ hrs/wk)	72.9%	280	75.6%	434	52.7%	112	<0.001
Hours worked per week, Mean (SD)	35.3 (13.4)	280	36.5 (12.7)	434	29.0 (14.7)	112	<0.001
Patients seen per week, Mean (SD)	22.8 (15.3)	265	27.3 (18.6)	424	13.4 (13.2)	116	<0.001
Working in publicinstitution	1.0%	312	3.8%	509	0.8%	136	<0.001
Working in Greater Toronto Area	67.5%	246	78.0%	382	88.6%	105	<0.001
**Annual Self-Reported Income**							
Mean (SD)	$63,834 ($57,101)	218	$45,624 ($44,081)	311	$29,230 ($41,645)	76	<0.001
Median	$55,000		$40,000		$15,000		<0.001

^**a**^Categorical variables are reported by counts and percentages; the *Pearson chi-square test* was used to compare frequencies of these variables across the three professions.

*Analysis of variance* was used to compare the means of continuous variables across the three professions. Median incomes were compared using a *non-parametric median test*.

## Results

### Response rate and representativeness

The study’s overall response rate was 23.3% (n = 1205), with 21.9% of invited TCM/acupuncture practitioners (n = 656), 24.1% of naturopaths (n = 366), and 27.7% of homeopaths (n = 183) participating in the online survey. Among providers, the gender profiles of surveyed TCM/acupuncture and naturopathy practitioners, and country of training data for homeopaths and naturopaths, shown in Table 2, closely echo prior demographic data for these professions, as reported by their provincial regulators in 2018 [30–32]. The current study’s homeopathic dataset trends towards slightly over-representing female practitioners as compared to the profession as a whole **(**p = 0.02). Furthermore, while Ontario’s Chinese medicine regulator’s country of training data for 2018 are incomplete [30], the proportion of Canadian-trained, registered TCM/acupuncture respondents in the study (49.7%) is similar to a more complete population dataset reported by the regulator in 2014 (54.4%) [33].

### Demographics

Overall, the demographic profiles of the three groups, shown in Table 2, differed significantly from one another in multiple ways. Over three-quarters of respondent naturopaths and homeopaths identified as female, significantly more than within the TCM/acupuncture profession (57.9%, p<0.001). Naturopaths, with a mean age of 42.5 years, were significantly younger—by about a decade on average—than their counterparts in the TCM/acupuncture and homeopathy professions (p<0.001). Related, a significantly lower proportion of naturopaths (12.2%) were under age 55 than homeopaths (44.0%) and TCM / acupuncture practitioners (37.6%) respectively (p<0.001).

Whereas 96.9% of surveyed naturopaths had completed their professional trainings in Canada, almost one-third of homeopaths and half of TCM / acupuncture practitioners had received their primary professional trainings outside of Canada. As detailed in Table 3, most non-Canadian trained TCM/acupuncture practitioners had received their primary professional education in an East Asian country; homeopaths educated outside of Canada, by contrast, had primarily completed their training in Southern Asia.

**Table 3:** Breakdown of primary training sites by continental region.

	Naturopaths (N = 311)		TCM / Acupuncture Practitioners (N = 492)^a^		Homeopaths (N = 133)^a^	
	%	n	%	n	%	n
North America	97.7%	310	51.4%	253	72.2%	96
Eastern Asia	2.3%	1	44.1%	217		
South Asia			0.8%	4	21.9%	29
Europe & Russia			2.2%	11	4.5%	6
Other			1.6%	8	1.5%	2

^a^Total n>N as some TCM/Acupuncture Practitioners and Homeopaths identified multiple distinct continental

regional locations as their primary professional training sites.

As Table 1 shows, a significantly higher proportion of surveyed TCM/acupuncture practitioners (58.0%) and homeopaths (57.6%) than naturopaths (19.9%) spoke more than one language with patients (p<0.001). The representation of specific language groups spoken across the three professions is detailed in Table 4. In addition to English, East Asian languages (in particular Mandarin and Cantonese) were most frequently used among TCM/acupuncture practitioners; South Asian languages (in particular Hindi) were most frequently spoken by homeopaths. Aside from English and French, Canada’s two official languages, Spanish and other European languages were the most common non-English languages spoken by naturopaths.

**Table 4:** Breakdown of languages spoken in clinical practice^a^.

	TCM / Acupuncture Practitioners (N = 470)		Naturopaths (N = 282)		Homeopaths (N = 125)	
	n	%	n	%	n	%
English	466	99.2%	282	100%	125	100%
French	22	4.7%	26	9.2%	12	9.6%
Chinese (Mandarin and/or Cantonese)	214	45.5%	4	1.4%	0	0
Other East Asian languages	23	4.9%	0	0	0	0
South Asian language(s)	6	1.3%	2	0.7%	34	27.2%
Eastern European / Russian language(s)	17	3.6%	8	2.8%	12	9.6%
Other European languages	9	1.9%	14	5.0%	16	12.8%
Other	8	1.7%	8	2.8%	4	3.2%

^a^Total n>N across all groups as some respondents reported using more than one language / language group in clinical practice.

### Practice characteristics

While three-quarters of surveyed TCM practitioners, and almost three-quarters of naturopaths reported working full-time in their profession, a significantly smaller proportion (p<0.001) of homeopaths in the study (52.7%) reported doing so (Table 1). Related, homeopaths reported working significantly fewer weekly hours on average than TCM/acupuncture practitioners (p<0.001) or naturopaths (p<0.001). The weekly average number of patient visits reported by TCM/acupuncture practitioners reported is furthermore twice that reported by homeopaths (p<0.001). Across all three groups, few practitioners reported working in public health care institutions as compared to private clinical practice settings. Most, however, reported working in the Greater Toronto Area, Ontario’s most populous region.

### Annual income

As shown in Table 2, self-reported mean annual incomes vary significantly between the three professions, with naturopaths reporting higher earnings on average ($63,834 SD $57,101) than TCM/acupuncture practitioners and homeopaths (p<0.001). Mean and median annual self-reported incomes across the professions, broken down by part- and full-time status, are presented in Table 5. Surveyed naturopaths working full-time reported earning over $20,000 more per year, on average, than their full-time TCM professional counterparts, and almost $30,000 more than the surveyed full-time homeopaths. Part-time homeopaths’ self-reported incomes are also substantially lower than in the other two professions; over 50% of Ontario’s part-time homeopaths reported earning less than $5000 per year in clinical practice.

**Table 5:** Income breakdown by part- and full-time status.

		Part-time (<30 hours/week)		Full-time (30+ hours/week)	
**Naturopaths** (n = 218)	*Mean*	$34,900 ($21,743)	50	$73,174 ($62,048)	159
	*Median*	$30,000		$60,000	
**TCM/Acupuncture Practitioners** (n = 311)	*Mean*	$24,322 (SD $16,535)	72	$52,018 ($48,700)	218
	*Median*	$23,500		$44,5000	
**Homeopaths** (n = 76)	*Mean*	$10,437 ($12,861)	35	$44,922 ($50,758)	44
	*Median*	$5,000		$27,500	

The study’s regression analysis (Table 6) shows that an inter-professional disparity in self-reported incomes persists among survey respondents after controlling for other variables, with TCM practitioners and homeopaths reportedly earning 32% and 39% less, respectively, than surveyed naturopaths (p<0.001). Across the three professions, the regression analysis furthermore shows that gender does not bear significantly on practitioners’ self-reported incomes after controlling for the number of hours worked and patients seen.

**Table 6:** Linear regression on log transformed income.

Independent Variables	Coefficients	95%	CI	p-value
*Profession type Ref*. *NAT*				
TCM	-0.394	-0.526	-0.262	<0.001
HOM	-0.491	-0.729	-0.254	<0.001
Female	-0.067	-0.210	0.077	0.362
Age	-0.012	-0.021	-0.003	0.011
# of years in practice	0.076	0.050	0.102	<0.001
# of years in practice squared	-0.002	-0.002	-0.001	<0.001
Trained outside of Canada	-0.447	-0.629	-0.264	<0.001
Hours worked per week, (log)	0.494	0.304	0.684	<0.001
Patients seen per week,(log)	0.506	0.370	0.641	<0.001
Work in a public institution	0.376	0.013	0.738	0.042
Working in Greater Toronto Area	0.072	-0.058	0.202	0.275
Constant	7.580	6.859	8.300	<0.001
N	521			
R squared	0.5288			

Notes. P-value in parenthesis were obtained with heteroskedastic robust standard errors.

Notably, being trained outside of Canada has a salient negative impact on surveyed practitioners’ self-reported incomes. Holding age, years of clinical experience and all other variables (including profession type) constant, practitioners trained outside of Canada report earning about 64 percent (exp-0.447 = 0.639) of the annual income of their Canadian-trained counterparts. Holding other variables constant, a reported income increase of about five percent is associated with both a ten percent increase in weekly hours worked and a ten percent increase in weekly patient visits. After controlling for all other variables, practising in a public health care institution is significantly associated with an increase in income.

## Discussion

In what follows, the authors provide a detailed discussion and interpretation of the study findings, with reference to relevant inter- and intra-jurisdictional comparator data. This section begins by discussing the demographic variables of gender, age and years in practice, country of training, and languages spoken, before turning to practice characteristics and income-related findings. With reference to the study aims, the authors draw attention to areas in need of additional research, as well as limitations within the present study.

### Gender

Ontario’s naturopathy, homeopathy and TCM/acupuncture professions are all predominantly comprised of female-identified practitioners, broadly consistent with prior reports of these T&CM occupational groups in other industrialized countries (Table 1). The degree of feminization evident in Ontario’s naturopathy profession is, however, slightly lower than that in Germany (16), Switzerland (14) and Australia (17), with an apparent global trend towards increased occupational feminization over the last fifteen years. The moderate occupational feminization of TCM/acupuncture practitioners in our study is similar to that reported in Australian (21) and Swiss (14) studies but lower than reported within the American acupuncturist profession [22–24, 26]. Similarly, a smaller proportion of Ontario homeopaths in our study identified as female as compared to New Zealand homeopaths in a 2017 study [25].

With reference to the Ontario, Canada context, however, the studied occupations are notably among the province’s *least* feminized health occupational groups. Ontario’s midwifery, nursing, dietetics and speech language pathology professions are for example comprised of over 90% women [29]. However, respondent naturopathic and homeopathic groups show a similar level of feminization to another of Ontario’s T&CM professions: registered massage therapy (78.3% female). In terms of gender, the TCM/acupuncture profession is approximately on par with Ontario’s pharmacy profession (57.7% female) but considerably more feminized than another Ontario T&CM occupation, chiropractic (35.8% female).

In this light, the feminization of the studied T&CM professions appears less exceptional–and perhaps more occupationally-specific, in the same way that some biomedical professions (e.g., nurses) tend historically to be more strongly feminized. Prior studies show women to be over-represented among most T&CM practitioner groups—[14, 17–20, 22, 23, 25, 34–37]–with the notable exceptions of chiropractors and osteopaths, who tend to be pre-dominantly male [38–41]. It has been elsewhere hypothesized that the strong masculinization of the two latter occupational groups may be explained by their greater centralization of biomedical epistemology, and a lesser emphasis on talk-focused clinical encounters than other T&CM occupations [42]. Additional research will be needed to better explain the differences in gender makeup between T&CM occupational groups, and in relation to biomedical health professions more broadly.

### Age and years in clinical practice

Ontario’s naturopaths in our study are similar in age to those in prior Canadian [18], American [20], and Australian [15,19] studies but about three years younger on average than European naturopaths [14, 16]. The province’s TCM/acupuncture practitioners and homeopaths report being similar in age and years of clinical experience to their counterparts in other industrialized countries but, as in other studies, considerably older and with more years of clinical experience than naturopaths. Additional research will be needed to explore possible reasons for this demographic trend, which takes on additional salience when considered within the Ontario provincial context.

In Ontario, the average age of non-physician health professionals is 44 years, just slightly older than naturopaths in our study (42.5 years). Average clinician ages within Ontario’s other regulated T&CM professions—chiropractors (43 years) and registered massage therapists (39 years) are similarly close to the provincial mean. However, there are just three other occupational groups among Ontario’s two-dozen regulated Ontario health professions with a mean age comparable to that of TCM/acupuncture practitioners (51.5 years) and homeopaths (53.1 years) in our study: dentists (50 years), dental technologists (50 years) and psychologists (53 years). What factors may account for more advanced age and/or, alternately, greater professional longevity within these occupational outliers warrants further investigation.

### Country of training and languages spoken

Outside of the Ontario context, data regarding naturopaths’ and homeopaths’ countries of training and languages spoken are scant or absent. That being said, our study results regarding TCM/acupuncture practitioners notably echo similar workforce data from other jurisdictions (Table 1) that show a considerable proportion of such practitioners to have: a) received their primary professional training in an East Asian country; and, b) speak an East Asian language in clinical practice. The present study similarly finds a notable proportion of surveyed Ontario homeopaths to have: a) been educated in Southern Asia; and, c) to speak a South Asian language with patients. We interpret these training and linguistic variables as a rough proxy for clinicians’ immigrant status from the aforementioned regions. This interpretation is reasonable given that traditional East Asian medicine and acupuncture are regulated professions in China and other East Asian countries [7]; and, homeopathy is similarly regulated in both India and Pakistan [43].

As compared to other regulated Ontario health professions, the province’s TCM/acupuncture and homeopathy professions are also outliers in terms of the proportion of internationally-trained practitioners represented. Whereas 9.4% of all Ontario health professionals report having been trained outside of Canada or the United States [29], this proportion increases to 48.6% and 27.7% of TCM/acupuncture practitioners and homeopaths, respectively, in our study. Ontario’s pharmacist profession is the only other regulated health occupational group in the province with a comparably high proportion (34.7%) of internationally-trained members. Similarly, about half of Ontario’s homeopaths and TCM/acupuncture practitioners speak a language other than Canada’s official English and/or French with patients, whereas just 24.2% of Ontario health professionals do so overall.

Part of the Ontario government’s articulated mandate in regulating the TCM/acupuncture and homeopathy professions was to recognize some forms of T&CM care as instrumental within the “cultural heritage” of Ontarians who had emigrated to Canada within recent immigrant influxes [44]. This mandate, along with our study findings, reinforce the specific cultural importance of some (but not all) T&CM occupations within particular ethnic minority communities. Related, Ontario’s other T&CM professions–naturopathy (in our study), chiropractic and registered massage therapy are almost entirely North American-trained; and are considerably more likely to be mono-lingual English speakers than the provincial average [29]. As such, it is likely that the latter groups play less central roles than their TCM/acupuncture and homeopathic counterparts in providing culturally-responsive care within ethnic minority communities.

### Practice characteristics

Comparisons of the present study’s practice characteristics with data from earlier studies is challenging in light of: a) the paucity of prior data about the weekly hours worked by homeopaths and TCM/acupuncture practitioners in other industrialized jurisdictions; and, b) the wide range of related results in prior studies of naturopaths. In the Ontario context, however, it does not appear as though being a T&CM professional *per se* bears notably on weekly workload. While a substantially larger proportion of surveyed TCM/acupuncture practitioners (73.2%) and naturopaths (72.4%) in our study report working full-time than do the 52.6% of Ontario’s allied health professionals overall [29], the study’s surveyed homeopaths reflect the provincial average more closely (52.7% full-time). Variance is similarly evident in the proportion of full-time workers within the province’s other two T&CM professions: chiropractic (62%) and massage therapy (34%) [29].

The very small proportion of study respondents who report working within public healthcare institutions, however, closely mirrors similar data from Ontario’s chiropractors and massage therapists. This practice characteristic may initially appear in stark contrast to the 33.7% of Ontario’s allied regulated health professionals, overall, who work in (public) hospital settings. However, there are other biomedical professions in the province–such as dentistry and optometry–whose care, like that of T&CM professionals, is paid for by the individual or reimbursed by private rather than public insurance; and, over 98% of whose clinicians work outside of hospital settings. As such, the tendency for an Ontario health profession’s members to work in the private sector is likely best understood within the context of the province’s publicly-funded health care system, which does not provide coverage for the services of all types of care.

### Income

Prior studies of American licensed TCM/acupuncture practitioners report similar average incomes to those reported in the present study; and, there are no prior reports of homeopathic practitioner income in the literature. After adjusting for inflation (2%), naturopaths in our Ontario study report earning about $19,000 less on average (mean) than did Canadian naturopaths in a 2019 survey by the American Association of Accredited Naturopathic Medical Colleges (AANMC) [28]. However, our study’s adjusted median naturopathic income was just over $4000 lower than that reported in the AANMC report one year later. It is unclear what factor(s) may account for this variance given that Ontario’s clinicians comprise the country’s largest and longest-standing pool of naturopaths nationwide [18]. The same AANMC study also reported considerably higher incomes, in US dollars, among American licensed naturopaths.

While income-related data for Ontario’s allied health professions are not readily available, it would appear as though the three studied T&CM professions may have lesser earning power than other occupational groups in the province. The Canadian government’s population survey data from 2018 indicate that the median pre-tax income of “families and unattached individuals in Ontario” was $62,300 [45]. This is well above the self-reported median pre-tax incomes represented, in our study, across Ontario’s naturopathic ($55,000), TCM/acupuncture ($36,500) and—in particular—homeopathic ($15,000) professions. The *full-time* naturopathic median in our study ($60,000) approaches the Ontario median; but, those of *full-time* TCM/acupuncture practitioners ($43,500) and homeopaths ($28,000) fall well below it.

For the greater Toronto area, where most of our study respondents report working, Canada’s federal government situated the 2018 household poverty line at $42,101 [46]. It is evident that about half of Ontario’s full-time TCM/acupuncture practitioners, and well over half of the province’s full-time homeopaths may not be in a position to independently sustain a household on their professional incomes.

Our study finding regarding Ontario T&CM professions’ comparatively low earnings within the broader occupational ecology persists when contextualized alongside other published data. According to recent Canadian government data, Ontario’s most-established group of T&CM professionals–chiropractors [47]—report a median annual income of $58,987 [48]: just above the median income of naturopaths in our study but below the provincial median income across all occupations. A 2018 study of Ontario’s *acupuncture-practising* registered massage therapists also reported a similar mean annual income of $55,295 [49]. If there is a single common occupational trait across Ontario’s T&CM occupations, it would appear to be their lower-than-average annual incomes within the overall provincial workforce.

Comparator data for the province’s allied health professions would prove useful in future for additional contextualization. Regardless, it remains evident that significant economic stratification exists across Ontario’s regulated health care professionals, exemplified most dramatically by the median annual income of $219,978 recently reported by the province’s biomedical physicians [48].

It would be worth interrogating in future what factors may be contributing to the comparatively low earnings of the province’s T&CM professions, in addition to the fact that these occupations work outside of the provincial public health care reimbursement system. One factor warranting exploration is the impact of these groups’ socio-cultural marginality—in particular for homeopaths, the plausibility of whose occupational epistemology is widely contested by biomedical scientists [43]. Another potential income-relevant factor warranting exploration is the availability of third-party insurance reimbursement for these professions’ services.

Of notable significance in our study was a finding that, independent of other factors, internationally-trained T&CM clinicians earned over one-third less per year, on average, than their Canadian-trained counterparts. This finding is consistent with previous reports of immigrants–in particular ethnic minority immigrants of colour, including South- and East-Asian immigrants–being notably disadvantaged in terms of workforce earnings in Canada [50]. However, to what degree various possible factors may be at play–including racism, linguistic barriers, differential price-setting, as well as the possible human capital advantage that immigrant clinicians may enjoy within their own ethnic communities, warrants further investigation.

### Study limitations

All study data were collected via respondents’ self-reporting and may thus be impacted by recall bias, or by social desirability bias among members of three occupational groups known to be pursuing enhanced socioeconomic stature in the jurisdiction under study. With a low overall response rate of 23.3%, it is possible that study results are not fully representative of the professions studied. The study team received some informal feedback from the occupations under study during the period when the survey was live that some practitioners were hesitant to participate in the study due to mistrust of academic research. Furthermore, it should be noted that the response rate, across professions, to the study’s income-related questions was lower than for most other questions; as such, the study’s income-related findings should best be confirmed in future studies.

## Conclusions

Echoing prior workforce studies of diverse T&CM practitioner groups, the demographic and practice characteristics of the three studied occupations are significantly distinct (p<0.001) on all measured datapoints. This finding affirms that it is, on the whole, inappropriate to use workforce data from one T&CM occupation to predict workforce characteristics for another. With a few minor exceptions, the workforce traits for each of the studied groups substantially mirror the characteristics of the same T&CM occupations in other industrialized countries (Table 1). This finding suggests that overall, workforce data for naturopaths, homeopaths and TCM/acupuncture practitioners from one industrialized jurisdiction may be reasonably considered a rough proxy for similar practitioners in another jurisdiction.

Additional research will be needed to establish the range of factors that may account for the stronger feminization of some T&CM occupations than others; and for the present study’s finding that Ontario’s homeopaths and TCM/acupuncture practitioners are, on average, considerably older than almost all other Ontario regulated health professions. Future explorations of the role of the two latter professions in delivering culturally-responsive health care within South–and East-Asian ethnic communities is also warranted, in light of the high proportion of internationally-trained, multilingual practitioners within these groups. Finally, it will be important to establish whether the relatively low self-reported incomes of the studied T&CM professional groups within Ontario’s broader workforce ecology is a trait that persists across other jurisdictions–in particular for internationally-trained (immigrant, ethnic minority) clinicians.

In conclusion, this study’s contribution is to advance workforce-related research regarding T&CM occupations in industrialized countries by positioning related data within its inter- and intra-jurisdictional contexts. In particular, additional earnings-related research is needed.
